# Validation of quantitative loop-mediated isothermal amplification assay using a fluorescent distance-based paper device for detection of *Escherichia coli* in urine

**DOI:** 10.1038/s41598-023-46001-6

**Published:** 2023-10-31

**Authors:** Natkrittaya Saengsawang, Panthita Ruang-areerate, Nuanlaong Kaeothaisong, Saovanee Leelayoova, Mathirut Mungthin, Piraporn Juntanawiwat, Patomroek Hanyanunt, Patsanun Potisuwan, Piyanate Kesakomol, Pornphan Butsararattanagomen, Pattarawadee Wichaiwong, Wijitar Dungchai, Toon Ruang-areerate

**Affiliations:** 1 Analytical Chemistry, Department of Chemistry, Faculty of Science, King Mongkut’s University of Technology Thonburi, Bangkok, 10140 Thailand; 2 BIOTEC, National Science and Technology Development Agency (NSTDA), Pathum Thani, 12120 Thailand; 3 Department of Parasitology, Phramongkutklao College of Medicine, Bangkok, 10400 Thailand; 4 Division of Microbiology, Department of Clinical Pathology, Phramongkutklao Hospital, Bangkok, 10400 Thailand; 5 Department of Microbiology, Phramongkutklao College of Medicine, Bangkok, 10400 Thailand

**Keywords:** Urinary tract infection, Applied microbiology, Bacterial techniques and applications

## Abstract

Uropathogenic *Escherichia coli* (UPEC) causes up to 90% of urinary tract infections (UTI) which is more prevalent among females than males. In urine, patients with symptomatic UTI usually have a high concentration of bacterial infection, ≥ 10^5^ colony-forming units (CFU) per mL, in which the culture method is regularly the gold standard diagnosis. In this study, a simple and inexpensive distance-based paper device (dPAD) combined with the fluorescent closed tube LAMP assay was validated for simultaneously screening and semi-quantifying the infection level of *E. coli* in 440 urine samples of patients with UTI. The dPAD could measure the LAMP amplicons and semi-quantify the levels of *E. coli* infection in heavy (≥ 10^4^ CFU/mL), light (≤ 10^3^ CFU/mL) and no infection. The sensitivity and specificity had reliable performances, achieving as high as 100 and 92.7%, respectively. The one step LAMP assay could be performed within 3 h, which was 7.5 times faster than the culture method. To empower early UTI diagnosis and fast treatment, this inexpensive dPAD tool combined with the fluorescent closed tube LAMP assay is simple, reliably fast and practically portable for point-of-care settings, particularly in resource-limited areas, which can be set up in all levels of healthcare facilities.

## Introduction

*Escherichia coli* (*E. coli*), a gram-negative bacterium, is a normal commensal intestinal microbiota in humans and animals^1,2^. In general, *E. coli* is nonpathogenic among humans but some strains cause diarrhea and urinary tract infection (UTI). Uropathogenic *E. coli* (UPEC) causing most UTIs, up to 90%, are considered the predominant cause of both community and nosocomial UTIs^3–5^ in which UPEC is responsible for 80% of uncomplicated UTIs among healthy women^6–8^. The prevalence of UTI is more common among females than males^9,10^, carries a high risk of bacterial infection among children^11^ and has recurrent bladder infection within 6 months after an initial episode in 20% to 30% of women^12^. Fecal flora is the source of UPEC spreading from the perineal, vaginal and peri-urethral areas to the lower urinary tract, where colonies could be established^13–16^. Lower UTI is referred to as acute cystitis, with symptoms, e.g., dysuria and an increased frequency of urination, while upper UTI can potentially lead to bacteremia, causing symptoms, e.g., flank pain, fever and malaise^17^. Frequently, UTI is difficult to treat and can cause parenchymal damage, leading to renal insufficiency and further complications^18–20^. Typically, patients with symptomatic UTI have a high concentration of bacterial infection, usually, ≥ 10^5^ colony-forming units (CFU) per mL as measured in a urine specimen^21–23^.

The gold standard laboratory diagnosis is regularly based on the conventional culture method that isolates and counts the colonies of interesting microorganisms such as *E. coli* present in infected urine using specific culture agar media^21–23^. Identifying colonies requires at least 24 h and takes around 5 to 7 days to confirm the report using biochemical and serological methods^3,24–27^. In contrast, molecular-based methods generally exhibit more sensitivity and specificity, for example, polymerase chain reaction (PCR) could specifically amplify genetic materials of the microorganisms less than 24 h, albeit, at comparatively higher cost regarding the requirement of expensive instruments^28–32^. Alternatively, different gene targets such as *lacZ*, *yaiO*, *uidA*, *fimH*, *hly*, *pap* and 16S rRNA genes could be selectively used and combined to detect *E. coli* in various condition^4,29,33^. Unlike PCR, loop-mediated isothermal amplification (LAMP) can generate a mixture of various lengths of stem-loop DNA amplicons under isothermal condition (60 to 65 °C) and synthesize large amounts of DNA in a short time^34,35^. Thus, LAMP is a user-friendly, low cost and simple approach requiring simple procedures and common thermal control instrument^36^. Therefore, the technique is feasible and valuable for all levels of hospitals and, especially, point-of-care settings in resource-limited areas to aid in diagnosing UTIs^37,38^. In addition, due to its simplicity, LAMP was possibly combined with various methods and devices to increase the sensitivity and feasibility, for example, magnetic separation^31^, microfluidic impedimetric sensing^31^, AuNP colorimetric precipitates^39,40^, semi-quantitative dPADs^38,41,42^ and microchips^43^.

In our related study, we successfully developed a semi-quantitative distance-based paper device (dPAD) using the fluorescent LAMP assay based on the SYBR safe dye indicator to detect *E. coli* in urine that could detect and semi-quantify *E. coli* in less than 2 h without the requirement of postreaction staining process^38^. A single drop of 4 µL LAMP reaction could quantify *E. coli* as low as 1 CFU/mL, whereas *E. coli* in natural urine could be semi-quantified in light and heavy infection levels, for which the cutoff value was 10^4^ CFU/mL^38^. However, the sensitivity and specificity of the dPAD were not completely evaluated for screening UTI and semi-quantifying *E. coli* in the natural urine of patients. Hence, in this study, we have validated a dPAD combined with the fluorescent closed tube LAMP assay to screen and evaluate *E. coli* infection levels in urine of patients with UTI. The study has proposed an inexpensive alternative method, faster than gold standard diagnosis, for screening and evaluating *E. coli* level among patients with UTI that is easily delivered to end-users, practically portable, cost effective and requires simple equipment, particularly in all levels of healthcare facilities to point-of-care settings in resource-limited areas.

## Results

### Fluorescent closed tube screening and semi-quantitative dPAD evaluation for *E. coli* infection in urine sample

**Figure 1:**
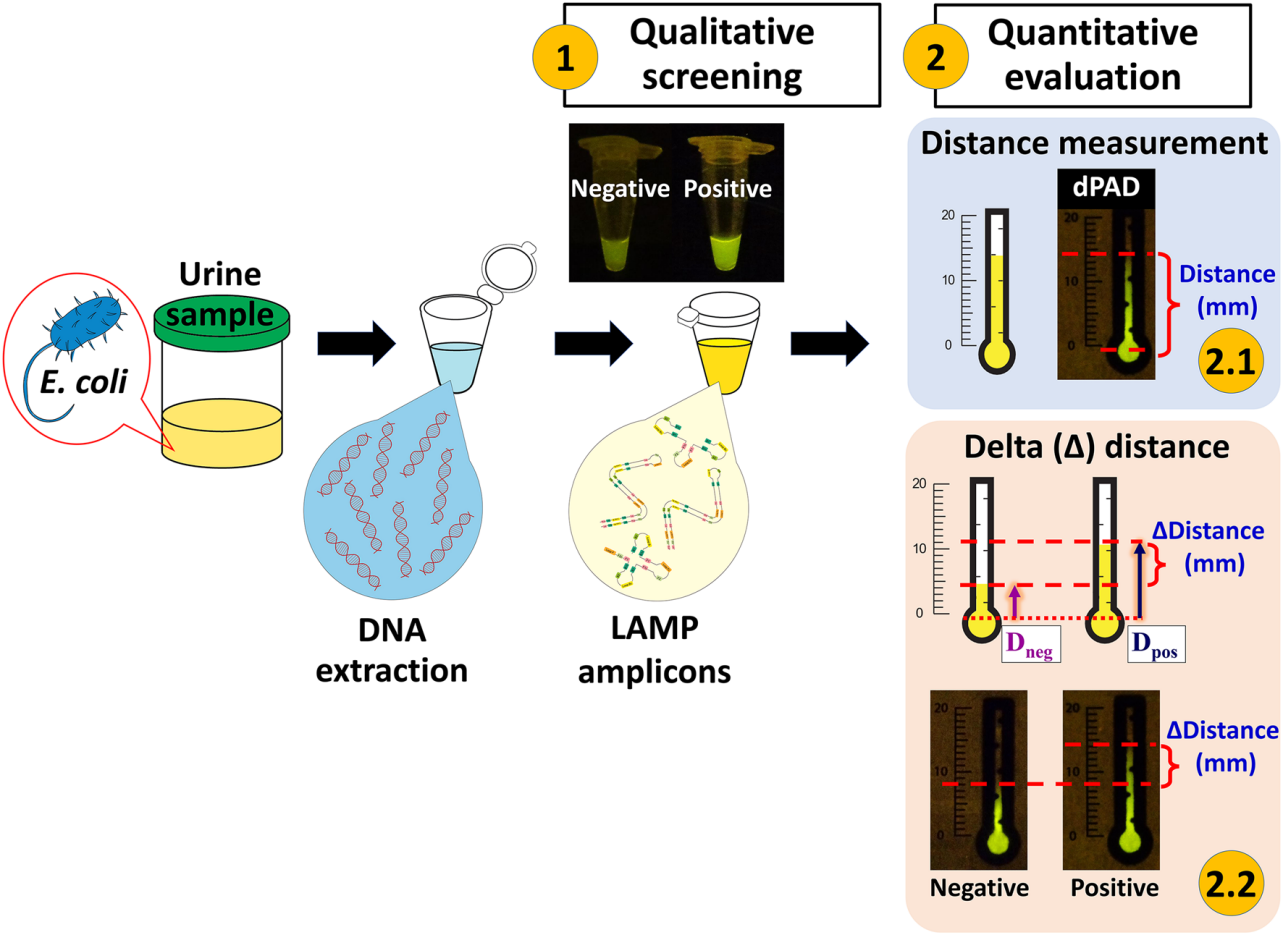
Schematic procedure of *E. coli* screening and quantitative evaluation in urine using distance-based paper device (dPAD) LAMP assay. Qualitative screening is the closed tube SYBR safe LAMP method that a positive result is the presence of fluorescene in a reaction tube under the BL transilluminator (1). For quantitative evaluation (2), the fluorescent migratory distance of dPAD (2.1) is directly measured in millimeters (mm), whereas delta distance-based paper device (ΔdPAD, 2.2) is the different distance (∆distance, mm) between negative control and sample (the distance of a sample; D_pos_, minus negative reference; D_neg_). The dPAD (2.1) combined with the fluorescent closed tube (1) result is examined and evaluated after the pre-reaction mixture of the fluorescent closed tube LAMP showed a positive result.

A schematic of the experimental principle to visualize LAMP products in pre-reaction detection using the fluorescent SYBR safe for rapid screening and in post-amplification detection using a dPAD to evaluate and semi-quantify LAMP amplicons is shown in Fig. 1. Qualitative screening could be performed using the closed tube SYBR safe LAMP method that is directly visualized by the naked eye under the BL transilluminator (1). To evaluate and semi-quantify the degree of *E. coli* infection in the urine sample, the dPAD can be employed by pipetting 4 µL of LAMP reaction of the closed tube SYBR safe LAMP method to the sample zone of the dPAD and waiting for 12 min (2). The migratory distance of the immobilized LAMP amplicons is measured directly by the naked eye under the BL transilluminator. The fluorescence of the migratory distance correlates to the final amounts of LAMP products (2.1). Otherwise, the difference in distance between the negative control using Milli Q water without genomic DNA and the sample (the distance of a sample minus negative reference as a cutoff value) or ΔdPAD is performed to standardize the distance of LAMP reaction mixture between each pre-reaction preparation (2.2). The process of detecting and immobilizing DNA on the cellulose paper owing to the physical adsorption and the charge interaction between DNA and the immobilized cationic polymers has been described by Saengsawang et al.^38^ and Ruang-areerate et al.^41^.

### Application of dPAD to screen and quantify *E. coli* infection in urine samples

**Figure 2:**
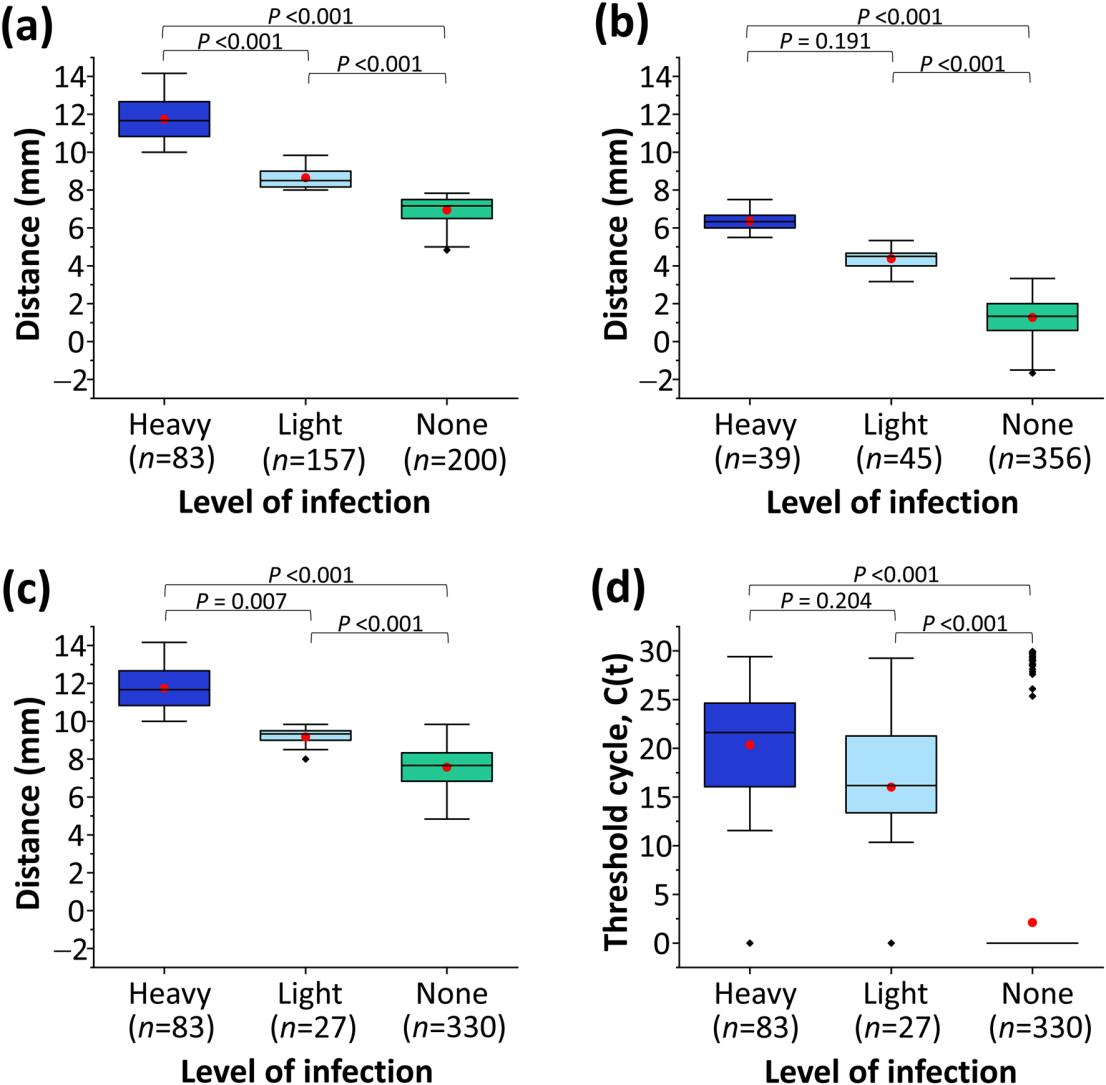
A boxplot representation of length variation of distance (mm) and threshold cycle; C(t), variation at each level of *E. coli* infection (heavy, light and none) using different detecting approaches for quantifying DNA amplicons, (**a**) distance-based paper device (dPAD), (**b**) Delta dPAD (ΔdPAD), (**c**) fluorescent closed tube combined with dPAD, (**d**) quantitative PCR (qPCR). Dark and light blue, and green boxplots represent heavy and light infection levels, and noninfection of *E. coli*, respectively. Each box's lower and upper boundaries are the 25th and the 75th percentile, respectively. Ranges are represented as bars (whiskers) below and above the box, and indicate the 10th and 90th percentiles, respectively. Mean values are indicated by the red dot marks on the boxplots.

Based on the cutoff value assigned by Saengsawang et al.^38^, we could divide the range of our migratory distance (millimeters; mm) in three categories: heavy (mean ± SD, 9.97 ± 0.73 mm; *n* = 6), light (7.95 ± 1.20 mm; *n* = 6) and none (4.58 ± 0.49 mm; *n* = 6) for measuring the fluorescent migratory distance in dPAD and ΔdPAD. Regarding the migratory distance immobilized by the device, the length of the dPAD (direct measurement), ΔdPAD (distance of a sample minus negative reference) and dPAD combined with the fluorescent closed tube (positive fluorescent closed tube with direct measurement of dPAD) could be semi-quantified and classified in three levels: the heavy (11.74 ± 1.14 mm, 6.35 ± 0.52 mm, 11.74 ± 1.14 mm; *n* = 83, 39, 83, respectively), light (8.65 ± 0.54 mm, 4.37 ± 0.55 mm, 9.17 ± 0.51 mm; *n* = 157, 45, 27, respectively) and none (6.95 ± 0.72 mm, 1.27 ± 1.09 mm, 7.57 ± 1.01 mm; *n* = 200, 356, 330, respectively) (Fig. 2). For two single detecting approaches (dPAD and ΔdPAD) and one combined detecting approach (dPAD combined with the fluorescent closed tube), significant differences were observed in the fluorescent migratory distance among three levels at a 95% confidence level (Kruskal*–*Wallis H test, *P*-value < 0.001, < 0.001 and < 0.001, respectively). However, the fluorescent migratory distances between heavy and light infected levels did not significantly differ in ΔdPAD and qPCR (heavy vs. light, Dunnett's test, *P*-value = 0.191, 0.204, respectively).

Among patients with UTI, an evaluation of the infection level was consistent in heavy infection of two single detecting approaches including qPCR and dPAD, and one combined detecting method, dPAD combined with the fluorescent closed tube. However, the error read-out of migratory distance was observed in light infection using dPAD (Fig. 2). The ΔdPAD showed how to solve the number of positive read-outs in light infection with a decrease of sensitivity, 81.0% (*n* = 68; 95% CI 70.9 to 88.7%), but increasing specificity, 95.5% (*n* = 340; 95% CI 92.8 to 97.4%) (Fig. 2b). According to simplified measurement and procedure, dPAD combined with the fluorescent closed tube approach was chosen to gain feasibility and highest sensitivity. The process could semi-quantify and evaluate the infection levels of *E. coli* among patients with UTI in heavy (*n* = 83), light (*n* = 27) and none (*n* = 330) (Fig. 2c). The qPCR was used to quantify the density degree of *E. coli* DNA at each infection level of the dPAD combined with the fluorescent closed tube approach in which mean values differed (heavy vs. light vs. none) by overlapping the 25th and the 75th percentile in heavy and light infections (Fig. 2d). Thus, the narrow C(t) was observed when limits of detection (LOD) using tenfold serial dilutions of *E. coli* DNA from 10^1^ to 10^6^ CFU/mL was performed (Fig. S1).

### Evaluation of sensitivity and specificity of semi-quantitative LAMP assay using dPAD

Four hundred forty DNA samples were extracted from urine including patients with *E. coli* (*n* = 84) and patients without (*n* = 356), to evaluate and validate the quantitative dPAD LAMP assay. Using the conventional culture method as a gold standard, the sensitivity and specificity of a combined detecting approach, the dPAD combined with the closed tube LAMP, were 100.0 (*n* = 84; 95% CI 95.7 to 100.0%) and 92.7% (*n* = 330; 95% CI 89.5 to 95.2%), respectively (Table 1). The sensitivity of two single detecting approaches including the fluorescent closed tube and dPAD was the same as dPAD combined with the closed tube LAMP, 100.0% (*n* = 84; 95% CI 95.7 to 100.0%). However, in qPCR and ΔdPAD; the sensitivity differed; 91.7 and 81.0% (*n* = 77, *n* = 68; 95% CI 83.6 to 96.6%, 70.9 to 88.7%, respectively). The specificity of the fluorescent closed tube, qPCR and ΔdPAD was high: 93.0, 93.8 and 95.5% (*n* = 331, 334, 340; 95% CI 89.8 to 95.4%, 90.8 to 96.1%, 92.8 to 97.4%, respectively), except dPAD, 56.2% (*n* = 200; 95% CI 50.9 to 61.4%).

**Table 1:** The sensitivity and specificity of quantitative LAMP assay using dPAD to detect *E. coli* DNA in urine of patients with UTI.

Molecular methods	No. (*n*) of samples with *E. coli* culture^a^		(%) Sensitivity (95%CI)	(%) Specificity (95%CI)	(%) PPV (95%CI)	(%) NPV (95%CI)
	Positive	Negative				
Qualitative detection						
Fluorescent closed tube^b^			100.0 (95.7–100.0)	93.0 (89.8–95.4)	77.1 (68.0–84.6)	100.0 (98.9–100.0)
Positive	84	25				
Negative	0	331				
Quantitative detection						
qPCR			91.7 (83.6–96.6)	93.8 (90.8–96.1)	77.8 (68.3–85.5)	97.9 (95.8–99.2)
Positive	77	22				
Negative	7	334				
dPAD^c^			100.0 (95.7–100.0)	56.2 (50.9–61.4)	35.0 (29–41.4)	100.0 (98.2–100.0)
Positive	84	156				
Negative	0	200				
ΔdPAD^d^			81.0 (70.9–88.7)	95.5 (92.8–97.4)	81.0 (70.9–88.7)	95.5 (92.8–97.4)
Positive	68	16				
Negative	16	340				
Qualitative and quantitative detection						
Fluorescent closed tube + dPAD^e^			100.0 (95.7–100.0)	92.7 (89.5–95.2)	76.4 (67.3–83.9)	100.0 (98.9–100.0)
Positive	84	26				
Negative	0	330				

^a^Total number of positive results was based on conventional culture method (gold standard diagnosis); ^b^SYBR safe was initially mixed in the reaction as a fluorescent indicator for qualitative screening; ^c^Measurement of fluorescent migratory distance was used to semi-quantify the LAMP amplicons; ^d^Delta distance of each sample (∆distance: a migratory distance of sample minus a migratory distance of negative control reference) was calculated following each LAMP preparation; ^e^Measurement of fluorescent migratory distance was used to semi-quantify the LAMP amplicons of positive LAMP reaction results of fluorescent closed tube screening.

The fluorescent closed tube, qPCR and dPAD combined with the fluorescent closed tube showed significant results that were in almost perfect agreement when compared with gold standard, conventional culture (*P*-value < 0.0001, < 0.0001, < 0.0001; Kappa = 0.84, 0.80, 0.83, respectively), whereas ΔdPAD was substantial (*P*-value < 0.0001; Kappa = 0.77) and, in dPAD, fair agreement was observed in (*P*-value < 0.0001; Kappa = 0.33). Interestingly, the dPAD combined with the closed tube LAMP was in almost perfect agreement when compared with the fluorescent closed tube and qPCR (*P*-value < 0.0001, < 0.0001; Kappa = 0.98, 0.92, respectively), whereas in fair agreement with ΔdPAD (*P*-value < 0.0001; Kappa = 0.83). In contrast, dPAD showed significant results with moderate agreement (*P*-value < 0.0001; Kappa = 0.44).

## Discussion

The validation of quantitative LAMP assay using dPAD was successfully achieved to quantify *E. coli* DNA in the urine of patients with UTI and evaluate the level of infection including heavy (≥ 10^4^ CFU/mL), light (≤ 10^3^ CFU/mL) and no infection levels (no *E. coli*) as described by Saengsawang et al.^38^. The highly sensitive LAMP assay could be selectively performed to screen and semi-quantify using the fluorescent SYBR safe and dPAD device, in the one-step LAMP method^41^. The assay could be simultaneously visualized for rapid screening using the fluorescent SYBR safe in pre-reaction detection. In contrast, the fluorescent migratory distance of dPAD was measured post-amplification detection to semi-quantify LAMP amplicons using DNA-immobilized cellulose Whatman No.1 and evaluate the degree of *E. coli* infection. The LAMP reaction could generate and increase a mixture of various lengths of stem-loop DNA amplicons depending on the amount of DNA precursor in each reaction sample that the stem-loop DNA products could be immobilized on the cellulose paper regarding the physical adsorption and covalent bonding between the amine-terminated nucleic acid and the polysaccharide^38^. Thus, the length of migratory distance is directly correlated with the number of LAMP products and reflects the initial *E. coli* concentration. The infection level was considered consistently in heavy infection of patients with UTI but not in light infection. The error read-out of migratory distance was probably due to a difference in the charge interaction between anionic DNA and cellulose Whatman No. 1 among each LAMP preparation.

Using ΔdPAD, the read-out of light infection was improved and certainly better than direct measurement using dPAD. However, reference subtraction was additionally required and impractically convenient. Therefore, the dPAD combined with the fluorescent closed tube was undoubtedly a suitable quantitative LAMP assay for evaluating and measuring the level of *E. coli* infection in the urine of patients with UTI because the method was feasibly inexpensive and practically convenient at all setting levels with high sensitivity (100.0%) and specificity (92.7%). Quantifying the negative results during the qualitative screening using the fluorescent closed tube LAMP is unnecessary in a pre-reaction mixture that, in controversy, the fluorescent migratory distance of these negative products could raise ambiguous measurement in the none and light infected samples due to the difference in the charge interaction among each LAMP preparation. Nonetheless, unclear negative samples could be measured using the dPAD to avoid unambiguous naked eye comparison^41^. Compared with qPCR, the assay did not require a set of serial CFU concentrations and a reference standard curve to measure the density of *E. coli* DNA.

The sensitivity (*n* = 84, 100.0%) and specificity (*n* = 330, 92.7%) of dPAD combined with the fluorescent closed tube LAMP assay were almost equivalent to those of single detecting approaches using qPCR (*n* = 77 and 334; 91.7 and 93.8%, respectively), the fluorescent closed tube (*n* = 84 and 331; 100.0 and 93.0%, respectively) and ΔdPAD (*n* = 68 and 340, 81.0 and 95.5%, respectively) for screening and quantifying *E. coli* DNA in urine of patients with UTI when conventional culture was used as the gold standard. Rather than single detecting approaches, the sensitivity of dPAD was equivalent (*n* = 84; 100.0%) but not for specificity (*n* = 200; 56.2%). Furthermore, the kappa statistical test indicated almost perfect agreement. In diagnosing parasitic and pathogenic infections such as strongyloidiasis, leishmaniasis, scrub typhus etc., relying on a single test alone for screening is inadequate ^44–46^. Likewise, the effective detection of *E. coli* could be rapidly determined by combined methods including enrichment time in the food matrix and PCR^47^, and using two specific markers, e.g., *uid*A and *usp*A^48^. Despite conventional methods, a biomimetic sensor method using a polyurethane layer on a stainless-steel chip and thermal resistance between the chip and liquid sensors could specifically detect and sensitively quantify the presence of *E. coli* from 10^2^ to 10^6^ CFU/mL^49^. Because of the nonrequirement of dPAD measurement of negative LAMP reaction results, the simplified combined detecting approach (fluorescent closed tube + dPAD) that is faster and less expensive is challenging.

In urine sample, patients with symptomatic UTI have typically a high concentration of bacterial infection, normally, ≥ 10^5^ CFU/mL^21–23^ that is consistent with the heavy infection level (≥ 10^4^ CFU/mL) in this study (Table 2). As described by Heytens et al.^33^, an increasing number of false negative diagnoses of UTI observed in the culture method when qPCR was used to test in the symptomatic group, suggesting the nucleic acid amplification tests (NAATs) could be more sensitive. The NAATs, such as PCR that requires costly instruments and well-trained lab technicians could specifically amplify specific gene regions of the microorganisms faster (2 to 4 h) than the gold standard diagnosis (> 24 h) or the conventional culture (Table 2)^28–32^ but the methods are usually qualitative, except qPCR. Nevertheless, the NAATs are limited and unsuitable for detecting all types of *E. coli* strains as effectively as the culture method. Additionally, nonrelated *E. coli* pathogens that contained an almost identical coding region of the housekeeping gene, such as 16S rRNA were prone to cross-amplification^38^. Unlike most multiple copies of housekeeping genes, the detection relied on *E. coli*’s specific genes, such as metabolic or transporter genes, leading to less sensitivity and prone to false-negative detection in low bacteria density^38^.

**Table 2:** The characteristic comparison of different *E. coli* detecting approaches among the culture (gold standard), LAMP assays (qualitative, quantitative and combined detecting LAMP assays) and PCR detection (qPCR and PCR) for diagnosis of UTI among patients.

Characteristics	Gold standard	PCR		LAMP			
	Culture	PCR	qPCR	Fluorescent closed tube	dPAD	ΔdPAD	Fluorescent closed tube + dPAD
Cost per reaction ($)^a^	1.21	4.43	5.61	4.00	4.23	4.46	4.23
Time (hrs, min)	24, 00	3, 30	3, 00	3, 00	3, 12	3, 12	3, 12
Validation							
Sensitivity (%)	(Reference)	91.7	91.7	100.0	100.0	81.0	100.0
Specificity (%)	(Reference)	93.8	93.8	93.0	56.2	95.5	92.7
Procedure requirement							
No laboratory	−	−	−	+	+	+	+
Noninstrument nucleic acid amplification (NINA)	−	−	−	+	+	+	+
Isothermal amplification	+	−	−	+	+	+	+
Fluorescent light source	−	−	+	+	+	+	+
Others							
Bacterial load	+	−	+	−	+	+	+
Detection limits range (CFU)	10^3^	−	−	−	10^−1^ to 10^3^	10^−1^ to 10^3^	10^−1^ to 10^3^
Field setting	−	−	−	+	+	+	+
Point-of-care (POC) setting	−	−	−	+	+	+	+

^a^The prices of reagents and DNA extraction kits were based on local supplies in Thailand.

Due to the requirement of non-costly instruments, dPAD combined with the fluorescent closed tube LAMP assay is convenient and affordable to local health service centers^41^ because a less experienced technician could employ the assay using step-by-step operating procedures^40^. Unlike the general LAMP assay, the dPAD required initial LAMP amplicons with an additional simplified process of migratory distance measurement to further quantify the infection level of *E. coli* among patients with UTI^38^. Based on the reagent prices in Thailand, the cost of the reaction mixture did not differ between PCR and LAMP, approximately US$4.5 per reaction, whereas qPCR cost about US$1 more (Table 2). However, the LAMP assay needs a simple heating device or noninstrument nucleic acid amplification (NINA) that is comparatively cheaper than PCR, especially qPCR^41,50^. Eventually, the dPAD combined with the fluorescent closed tube LAMP assay could measure the infection level of *E. coli* in the urine of patients with UTI within 3 h 12 min (7.5 times) faster than the conventional culture method (> 24 h) and cost ~ US$1.4 cheaper than qPCR. In the leishmaniasis study, DNA could be prepared within 10 min for short term preservation using a simplified boiling extraction method^51^, which is faster than those of DNA extraction kits (2 h) in this study. However, a centrifuge is required for pelleting the *E. coli* in urine.

In this present work, we have validated different quantitative LAMP approaches using dPAD for quantifying *E. coli* in the urine of patients with UTI and that the dPAD combined with the fluorescent closed tube LAMP assay was comparatively simple, showing high sensitivity (100.0%) and specificity (92.7%), and could selectively proceed with a simultaneous qualitative screening and semi-quantitative evaluation in a one-step LAMP assay without the requirement of advanced instruments (Table 2). However, the assay required a transilluminator to excite fluorescence as a trade-off for fast screening and semi-quantifying the infection level of UTI diagnosis^41^. Regarding the simplified detection of heavy, light and noninfection status, the dPAD remains limited. However, it remains sufficient for setting up at all levels of healthcare facilities, particularly in field diagnostics to point-of-care settings in resource-limited areas because the assay is reliably fast, simple, inexpensive and practical. Hence, the dPAD combined with a fluorescent closed tube LAMP assay is a promising rapid approach to facilitate early UTI diagnosis and promote suitable fast treatment that can reduce severity and prevent disease progression.

## Conclusion

We validated the simplified and inexpensive dPAD combined with the fluorescent closed tube LAMP assay to screen and semi-quantify the infection level of *E. coli* in the urine of 440 patients with UTI. The single quantitative detecting approaches showed less sensitivity in ΔdPAD (81.0%) and less specificity in dPAD (56.2%) when compared with the combined detecting approach (fluorescent closed tube + dPAD) (100.0 and 92.7%, respectively). The additional step of semi-quantitative immobilized dPAD in post-amplification preparation of the closed tube LAMP assay required 12 min to measure the level of infection. In conclusion, the one-step semi-quantitative dPAD combined with the fluorescent closed tube LAMP assay is reliably fast, selectively practical and suitable for simultaneous screening and semi-quantifying *E. coli* in urine as an alternative method in addition to the conventional culture, especially in healthcare facilities with limited resources that efficiently deliver services to end-users.

## Materials and methods

### Ethics statement

Leftover urine samples, routinely collected for UTI diagnostic purposes, were stored in a microbiological hospital laboratory. Samples were coded and anonymized before specimen processing and analysis. Relevant guidelines and regulations were carried out using all methods. The study was reviewed and approved by the Institutional Review Board, Royal Thai Army Medical Department (IRBRTA 1427/2564 and IRBRTA 1214/2565). The Institutional Review Board, Royal Thai Army Medical Department (IRBRTA) waived the need of obtaining informed consent.

### Materials and reagents

The reagents and enzyme included Millipore Milli Q water system (R ≥ 18 MΩ cm), magnesium sulfate (New England Biolabs, Ipswich, MA, USA), 1× Thermopol buffer (New England Biolabs), Betaine (Sigma-Aldrich, St. Louis, MO, USA), SYBR™ Safe DNA Gel Stain (Thermo Fisher Scientific, Waltham, MA, USA), dNTP mix solution (Promega Corporation, Madison, WI, USA), *Bst* DNA Polymerase Large Fragment (New England Biolabs) and KAPA SYBR^®^ FAST (Sigma-Aldrich). The chemical solutions were prepared and made according to Saengsawang et al.^38^. The tissue Genomic DNA Extraction Mini Kit was purchased from Favorgen^®^ Biotech Corp. (Ping Tung, Taiwan). The dPADs were fabricated from Whatman No. 1 (Vernon Hills, IL, USA), according to Saengsawang et al.^38^.

### *E. coli* culture and urine sample preparation

*Escherichia coli* isolate (ATCC 25922) was cultured and the colony-forming units (CFU) were calculated per mL using 0.5 McFarland Equivalence Turbidity standard (Remel, Lenexa, KS, USA) to obtain an equivalent concentration of *E. coli* to 1.5 × 10^8^ CFU/mL as described previously^38^. The culture solutions were centrifuged at 2300*g* for 10 min to collect *E. coli* pellets and stored at − 20 °C.

For urine, the samples were spontaneously mixed and inoculated using a calibrated loop on McConkey agar or blood agar. The agar plate was incubated at 37 °C for 24 h, and then CFU per mL of bacteria was measured based on colony counts in which the UTI cutoff value was typically ≥ 10^5^ CFU/mL. On the other hand, ten milliliters of leftover urine sample was centrifuged to precipitate *E. coli* at 2300*g* for 10 min and washed three times with phosphate buffer saline. The pellets were stored at − 20 °C before DNA extraction. Of 440 uroculture, 84 (19.1%) were *E. coli* and 86 (19.6%) were other uropathogens.

### DNA extraction and qPCR amplification

The total DNA of the pellets of *E. coli* culture and urine sample were extracted using the FavorPrep™ Tissue Genomic DNA Extraction Mini Kit (Favorgen® Biotech Corp.) according to manufacturer protocol. The extracted DNA was eluted to 100 µL final volume and maintained at − 20 °C until used. A ten fold dilution of DNA was used as a DNA template for qPCR and LAMP amplification.

The qPCR amplification was performed under a final volume of 10 µL consisting of a 1 µL DNA template, 2 pmol of each primer (F3; 5′-GCTTCTTTGCTGACGAGTGG and B3; 5′-TCAGACCAGCTAGGGATCG) and 5 µL of KAPA SYBR^®^ FAST (Sigma-Aldrich). The highly conserved 16S ribosomal RNA gene of *E. coli* was amplified using a CFX96™ Real-Time PCR Detection System (Bio-Rad, Hercules, CA, USA) with *E. coli* isolate’ DNA (ATCC 25922) as a positive control, whereas the negative control was Milli Q water. The qPCR profile was one cycle of initial amplification at 95 °C for 3 min, followed by 45 cycles of amplification: 10 s denaturation at 95 °C and 30 s extension at 60 °C.

### LAMP reaction and semi-quantitative dPAD LAMP assay

The LAMP reaction was prepared in a total volume of 25 µL of reaction mixture to amplify the highly conserved 16S ribosomal RNA gene of *E. coli* as described by Saengsawang et al.^38^. In brief, each reaction mixture consisted of 1× Isothermal Amplification Buffer (New England Biolabs), 6 mM MgSO_4,_ 0.8 M Betaine (Sigma-Aldrich), 1.4 mM dNTP (Promega Corporation), 1.6 µM of each inner primer (FIP and BIP, Table S1), 0.4 µM of each outer primer (F3 and B3, Table S1), 8 U of *Bst* DNA Polymerase Large Fragment (New England Biolabs) and 2 µL of template DNA. In addition to closed tube LAMP detection, 1 × SYBR™ Safe DNA Gel Stain (Thermo Fisher Scientific) was added to each reaction mixture^52^ as the nonspecific fluorescent dye indicator of LAMP products. The reaction mixture was incubated at 65 °C for 60 min and inactivated the reaction at 80 °C for 2 min. A negative control using Milli Q water without genomic DNA sample was included in all LAMP amplifications. Each LAMP reaction was divided in two portions: one for qualitative fluorescent closed tube screening and the other for semi-quantitative dPAD evaluation.

For qualitative SYBR safe closed tube LAMP screening, the target LAMP amplicons were examined based on direct visual inspection of the reaction tubes using either a blue light (BL) or an ultraviolet transilluminator^40,52,53^. A positive amplification showed vivid fluorescent emission, whereas no fluorescence emission was detected in the absence of amplification. Five microliters of reaction mixture were electrophoresed on 1.5% agarose gel and visualized using Molecular Imager^®^ Gel Doc™ XR+ System with Imager Lab^TM ^3.0 (Bio-Rad, Hercules, CA, USA) (Fig. S2).

To detect and semi-quantify *E. coli* on the dPAD, 4 µL of the LAMP reaction mixture was loaded on the sample zone in which the sample flowed in the detection zone and dried within 12 min at room temperature. The migratory distance of fluorescence was measured in millimeters (mm) by visual inspection under BL^38^. The dPADs were fabricated as described by Saengsawang et al.^38^.

### Evaluation of sensitivity and specificity of semi-quantitative LAMP assay using dPAD

The sensitivity and specificity of qualitative (fluorescent closed tube), quantitative (qPCR, dPAD and ΔdPAD), and combined detecting LAMP approaches (fluorescent closed tube + dPAD) were determined using a total of 440 genomic DNA samples from urine, consisting of 84 confirmed *E. coli* infection and 356 uninfected cases. Diagnosis of UTI was confirmed when *E. coli* cell culture on agar plate was positive, typically ≥ 10^5^ CFU/mL cutoff value. Agar plate culture results using McConkey or blood agar are a gold standard for UTI diagnosis^3,21–23,26,27^. These data were used to validate and evaluate the sensitivity and specificity of different LAMP approaches for detecting *E. coli* in the urine of patients with UTI. The strength of the agreement was determined between qualitative detection including the fluorescent closed tube (SYBR safe), quantitative detections using qPCR and dPAD LAMP detection in postamplification (dPAD and ΔdPAD), and combined pre-reaction and post-amplification detection (dPAD combined with the fluorescent closed tube). The methods were assessed using the kappa statistical test at 95% confidence intervals (CI), and a *P*-value < 0.05 was considered statistically significant using STATA, Version SE14 (Stata Corporation, College Station, TX, USA).

In contrast to the direct measurement of the fluorescent migratory distance of dPAD, delta distance-based paper device (ΔdPAD) was the delta distance of each sample (∆distance) that was the different distance between negative control and sample (the distance of a sample minus negative reference). Thus, the ΔdPAD value of the negative reference was zero, a cutoff value. For dPAD combined with the fluorescent closed tube (fluorescent closed tube + dPAD), the semi-quantitative result was examined and evaluated after the pre-reaction mixture of the fluorescent closed tube LAMP showed a positive result. When the fluorescent closed tube screening results were negative, the migratory distance of the reaction mixture would not be further measured by a semi-quantitative dPAD.

### Supplementary Information

Supplementary Information.

## Data Availability

The datasets generated during the current study are available from the corresponding author on reasonable request.

## References

[1] Foxman B (2010). The epidemiology of urinary tract infection. Nat. Rev. Urol..

[2] Kahlmeter G (2000). The ECO.SENS Project: A prospective, multinational, multicentre epidemiological survey of the prevalence and antimicrobial susceptibility of urinary tract pathogens—interim report. J. Antimicrob. Chemother..

[3] Alanazi M, Alqahtani F, Aleanizy F (2018). An evaluation of *E. coli* in urinary tract infection in emergency department at KAMC in Riyadh, Saudi Arabia: Retrospective study. Ann. Clin. Microbiol. Antimicrob..

[4] Dadi B (2020). Distribution of virulence genes and phylogenetics of uropathogenic *Escherichia coli* among urinary tract infection patients in Addis Ababa, Ethiopia. BMC Infect. Dis..

[5] Loh K, Sivalingam N (2007). Urinary tract infections in pregnancy. Malays. Fam. Phys..

[6] Shea A (2020). *Escherichia coli* CFT073 fitness factors during urinary tract infection: Identification using an ordered transposon library. Appl. Environ. Microbiol..

[7] Jancel T, Dudas V (2002). Management of uncomplicated urinary tract infections. West. J. Med..

[8] Hooton TM (2012). Uncomplicated urinary tract infection. N. Engl. J. Med..

[9] Foxman B (2014). Urinary tract infection syndromes: Occurrence, recurrence, bacteriology, risk factors, and disease burden. Infect. Dis. Clin. N. Am..

[10] Lipsky BA (1989). Urinary tract infections in men. Ann. Intern. Med..

[11] Wald E (2004). Urinary tract infections in infants and children: A comprehensive overview. Curr. Opin. Pediatr..

[12] Scholes D (2000). Risk factors for recurrent urinary tract infection in young women. J. Infect. Dis..

[13] Moreno E (2006). Relationship between *Escherichia coli* strains causing urinary tract infection in women and the dominant faecal flora of the same hosts. Epidemiol. Infect..

[14] Chen SL (2013). Genomic diversity and fitness of *E. coli* strains recovered from the intestinal and urinary tracts of women with recurrent urinary tract infection. Sci. Transl. Med..

[15] Yamamoto S (1997). Genetic evidence supporting the fecal-perineal-urethral hypothesis in cystitis caused by *Escherichia coli*. J. Urol..

[16] Hooton TM (2001). Recurrent urinary tract infection in women. Int. J. Antimicrob. Agents.

[17] Kudinha, T. *Escherichia coli—Recent Advances on Physiology, Pathogenesis and Biotechnological Applications* Ch. 3, 45-69 (2017).

[18] Draghijeva E (2000). Virulence factors in *Escherichia coli* from children with pyelonephritis. Clin. Microbiol. Infect..

[19] Leroy S (2010). Impressive renal damage after acute pyelonephritis in a child. Pediatr. Nephrol..

[20] Kalra OP, Raizada A (2009). Approach to a patient with urosepsis. J. Glob. Infect. Dis..

[21] Kass EH (1962). Pyelonephritis and bacteriuria. A major problem in preventive medicine. Ann. Intern. Med..

[22] Wilson ML, Gaido L (2004). Laboratory diagnosis of urinary tract infections in adult patients. Clin. Infect. Dis..

[23] El-Mahdy R, Mahmoud R, Shrief R (2021). Characterization of *E. coli* phylogroups causing catheter-associated urinary tract infection. Infect. Drug Resist..

[24] Houpikian P, Raoult D (2002). Traditional and molecular techniques for the study of emerging bacterial diseases: One laboratory’s perspective. Emerg. Infect. Dis..

[25] Aguero-Rosenfeld Maria E (2000). Serology of culture-confirmed cases of human granulocytic ehrlichiosis. J. Clin. Microbiol..

[26] Elsayed A, Mohamedin A, Ata T, Ghazala N (2016). Molecular characterization of multidrug resistant clinical *Escherichia coli* isolates. Am. J. Biochem. Mol. Biol..

[27] Liu Y (2019). Detection of 12 common food-borne bacterial pathogens by TaqMan real-time PCR using a single set of reaction conditions. Front. Microbiol..

[28] Rajapaksha P (2019). A review of methods for the detection of pathogenic microorganisms. Analyst.

[29] Molina F (2015). Improved detection of *Escherichia coli* and coliform bacteria by multiplex PCR. BMC Biotechnol..

[30] Munkhdelger Y, Gunregjav N, Dorjpurev A, Juniichiro N, Sarantuya J (2017). Detection of virulence genes, phylogenetic group and antibiotic resistance of uropathogenic *Escherichia coli* in Mongolia. J. Infect. Dev. Ctries.

[31] Sharif S (2019). A novel impedimetric sensor for detecting LAMP amplicons of pathogenic DNA based on magnetic separation. Sens. Actuators B Chem..

[32] Saharan P, Dhingolia S, Khatri P, Duhan J, Gahlawat S (2014). Loop-mediated isothermal amplification (LAMP) based detection of bacteria: A review. Afr. J. Biotechnol..

[33] Heytens S (2017). Women with symptoms of a urinary tract infection but a negative urine culture: PCR-based quantification of *Escherichia coli* suggests infection in most cases. Clin. Microbiol. Infect..

[34] Notomi T (2000). Loop-mediated isothermal amplification of DNA. Nucleic Acids Res..

[35] Itou, T., Markotter, W. & Nel, L. H. *Current Laboratory Techniques in Rabies Diagnosis, Research and Prevention* Vol. 1 (Rupprecht, C. & Nagarajan, T.) 85–95 (Academic Press, 2014).

[36] Notomi T, Mori Y, Tomita N, Kanda H (2015). Loop-mediated isothermal amplification (LAMP): Principle, features, and future prospects. J. Microbiol..

[37] Li Y, Fan P, Zhou S, Zhang L (2017). Loop-mediated isothermal amplification (LAMP): A novel rapid detection platform for pathogens. Microb. Pathog..

[38] Saengsawang N (2020). Development of a fluorescent distance-based paper device using loop-mediated isothermal amplification to detect *Escherichia coli* in urine. Analyst.

[39] Suwannin P (2021). Heat-enhancing aggregation of gold nanoparticles combined with loop-mediated isothermal amplification (HAG-LAMP) for *Plasmodium falciparum* detection. J. Pharm. Biomed. Anal..

[40] Ruang-areerate T (2021). Development of loop-mediated isothermal amplification (LAMP) assay using SYBR safe and gold-nanoparticle probe for detection of *Leishmania* in HIV patients. Sci. Rep..

[41] Ruang-areerate T (2022). Distance-based paper device using combined SYBR safe and gold nanoparticle probe LAMP assay to detect *Leishmania* among patients with HIV. Sci. Rep..

[42] Hongwarittorrn I, Chaichanawongsaroj N, Laiwattanapaisal W (2017). Semi-quantitative visual detection of loop mediated isothermal amplification (LAMP)-generated DNA by distance-based measurement on a paper device. Talanta.

[43] Zhang M (2021). A newly developed paper embedded microchip based on LAMP for rapid multiple detections of foodborne pathogens. BMC Microbiol..

[44] Keiser Paul B, Nutman Thomas B (2004). *Strongyloides*
*stercoralis* in the immunocompromised population. Clin. Microbiol. Rev..

[45] Pandey N (2018). Detection of *Leishmania* DNA in saliva among patients with HIV/AIDS in Trang Province, southern Thailand. Acta Trop..

[46] Ruang-areerate T (2011). Genotype diversity and distribution of *Orientia** tsutsugamushi* causing scrub typhus in Thailand. J. Clin. Microbiol..

[47] Choi Y (2018). Rapid detection of *Escherichia coli* in fresh foods using a combination of enrichment and PCR analysis. Korean J. Food Sci. An..

[48] Godambe LP, Bandekar J, Shashidhar R (2017). Species specific PCR based detection of *Escherichia coli* from Indian foods. 3 Biotech..

[49] Cornelis P (2019). Sensitive and specific detection of *E. coli* using biomimetic receptors in combination with a modified heat-transfer method. Biosens. Bioelectron..

[50] Curtis KA (2012). Isothermal amplification using a chemical heating device for point-of-care detection of HIV-1. PLoS ONE.

[51] Sriworarat C, Phumee A, Mungthin M, Leelayoova S, Siriyasatien P (2015). Development of loop-mediated isothermal amplification (LAMP) for simple detection of *Leishmania* infection. Parasit. Vectors.

[52] Thita T, Manomat J, Leelayoova S, Mungthin M, Ruang-areerate T (2019). Reliable interpretation and long-term stability using SYBR™ safe fluorescent assay for loop-mediated isothermal amplification (LAMP) detection of *Leishmania* spp. Trop. Biomed..

[53] Sukphattanaudomchoke C (2020). Simplified closed tube loop mediated isothermal amplification (LAMP) assay for visual diagnosis of *Leishmania* infection. Acta Trop..

