# Supplementary material for: Validation of quantitative loop-mediated isothermal amplification assay using a fluorescent distance-based paper device for detection of Escherichia coli in urine
Source: Sci Rep. 2023 Oct 31;13:18781. doi: 10.1038/s41598-023-46001-6 (PMC10618465; doi:10.1038/s41598-023-46001-6)
Supplement: Supplementary file 1 — Supplementary Information. [file 41598_2023_46001_MOESM1_ESM.pdf]

Validation of quantitative loop-mediated isothermal amplification assay using a fluorescent distance-based paper device for detection of *Escherichia coli* in urine

Natkrittaya Saengsawang[1], Panthita Ruang-areerate[2], Nuanlaong Kaeothaisong[3], Saovanee Leelayoova[3], Mathirut Mungthin[3], Piraporn Juntanawiwat[4], Patomroek Hanyanunt[4], Patsanun Potisuwan[4], Piyanate Kesakomol[5], Pornphan Butsararattanagomen[1], Pattarawadee Wichaiwong[1], Wijitar Dungchai[1*] and Toon Ruang-areerate[3*]

[1]Analytical Chemistry, Department of Chemistry, Faculty of Science, King Mongkut's University of Technology Thonburi, Bangkok, 10140, Thailand. [2]BIOTEC, National Science and Technology Development Agency (NSTDA), Pathum Thani, 12120, Thailand. [3]Department of Parasitology, Phramongkutklao College of Medicine, Bangkok, 10400, Thailand. [4]Division of Microbiology, Department of Clinical Pathology, Phramongkutklao Hospital, Bangkok, 10400, Thailand. [5]Department of Microbiology, Phramongkutklao College of Medicine, Bangkok, 10400, Thailand.

*Address correspondence to Toon Ruang-areerate, Department of Parasitology, Phramongkutklao College of Medicine, Bangkok, 10400, Thailand; Wijitar Dungchai, Department of Chemistry, Faculty of Science, King Mongkut's University of Technology Thonburi, Bangkok, 10140, Thailand. E-mails: youangtr@yahoo.com; wijitar.dun@kmutt.ac.th

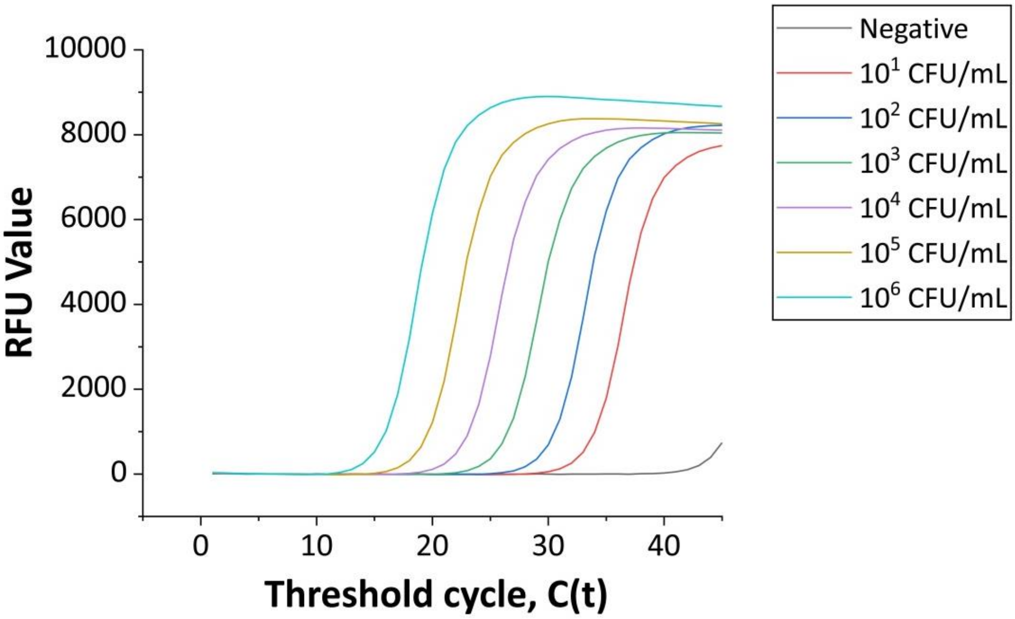

Figure S1. The real time amplification curves of serial concentration of *E. coli* DNA at 10^1 to 10^6 CFU/mL using quantitative PCR.

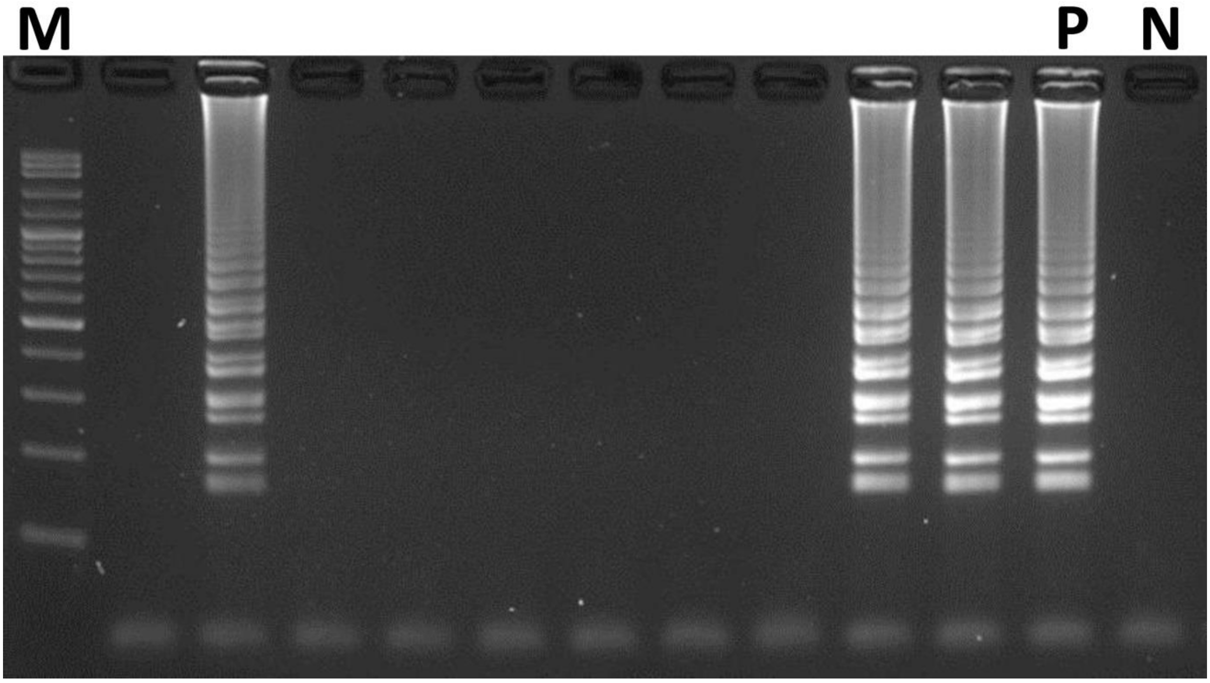

Figure S2. The various multiple bands of stem-loop DNA structure of LAMP amplification based on 16S ribosomal RNA gene of *E. coli* from urine of patients. Abbreviations of variable names: 100 bp ladder (M), positive control (P), negative control (N).

Primer	Sequence (5′−3′)	Length (bp)
F3	GCT TCT TTG CTG ACG AGT GG	20
B3	TCA GAC CAG CTA GGG ATC G	19
FIP	TTG GTC TTG CGA CGT TAT GCG GAT GTC TGG GAA ACT GCC TG	41
BIP	GGC CTC TTG CCA TCG GAT GTC GCC TAG GTG AGC CGT TA	38

Table S1. LAMP primer sequences (Saengsawang et al., 2020) used in this study.
